# Integrated Approaches and Empirical Models for Investigation of Parasitic Diseases in Northern Wildlife

**DOI:** 10.3201/eid1401.071119

**Published:** 2008-01

**Authors:** Eric P. Hoberg, Lydden Polley, Emily J. Jenkins, Susan J. Kutz, Alasdair M. Veitch, Brett T. Elkin

**Affiliations:** * US Department of Agriculture Agricultural Research Service, Beltsville, Maryland, USA; † University of Saskatchewan Western College of Veterinary Medicine, Saskatoon, Saskatchewan, Canada; ‡ Environment Canada, Saskatoon, Saskatchewan, Canada; § University of Calgary Faculty of Veterinary Medicine, Calgary, Alberta, Canada; ¶ Government of the Northwest Territories, Norman Wells, Northwest Territories, Canada; # Government of the Northwest Territories, Yellowknife, Northwest Territories, Canada

**Keywords:** host-parasite relationships, disease ecology, epidemiology, northern North America, archives, biological models, wildlife, climate change, International Polar Year, perspective

## Abstract

The North is a frontier for exploration of emerging infectious diseases and the large-scale drivers influencing distribution, host associations, and evolution of pathogens among persons, domestic animals, and wildlife. Leading into the International Polar Year 2007–2008, we outline approaches, protocols, and empirical models derived from a decade of integrated research on northern host–parasite systems. Investigations of emerging infectious diseases associated with parasites in northern wildlife involved a network of multidisciplinary collaborators and incorporated geographic surveys, archival collections, historical foundations for diversity, and laboratory and field studies exploring the interface for hosts, parasites, and the environment. In this system, emergence of parasitic disease was linked to geographic expansion, host switching, resurgence due to climate change, and newly recognized parasite species. Such integrative approaches serve as cornerstones for detection, prediction, and potential mitigation of emerging infectious diseases in wildlife and persons in the North and elsewhere under a changing global climate.

## Abstract 2

A decade of research has yielded a multidisciplinary approach for detection, prediction, and potential mitigation measures.

Insights about environmental change and emerging infectious disease have been derived primarily from temperate and tropical systems (*1*–*3*), even though host–pathogen relationships at higher latitudes of the Northern Hemisphere are affected by rapid climate change and anthropogenic disturbance (*4*–*8*). The North, therefore, serves as a sentinel and a complementary window, relative to temperate and tropical environments, for assessing the cascading ecologic effects of global climate change. Within relatively simple northern ecosystems, it is possible to differentiate signals of climate change from those associated with nonclimatologic drivers. Consequently, the North is a vital frontier for the exploration of biotic, abiotic, and historical determinants that influence the distribution, host associations, and evolution of pathogens in wildlife and human populations (*4*,*6*,*9*,*10*).

Host–parasite systems are particularly sensitive indicators of climate change and other causes of ecologic perturbation (*3*,*11*). Many macroparasites (e.g., helminths and arthropods) undergo life cycles with free-living stages whose development and survival are influenced by temperature, among other abiotic factors (*2*,*6*). For example, small changes in absolute temperatures can have substantial effects on the transmission dynamics of protostrongylid lungworms and muscleworms (species of *Parelaphostrongylus*, *Protostrongylus*, and *Umingmakstrongylus*), which cycle among the environment, gastropod (slug and snail) intermediate hosts, and ungulate (caribou, muskoxen, thinhorn sheep, moose) definitive hosts (Figure 1). These parasites are pathogenic in wildlife important to northern communities for cultural, economic, and spiritual reasons (*4*). The importance of keystone wildlife (those critical for the function and continuity of ecosystems and northern peoples), the potential sensitivity of host–pathogen assemblages to environmental disturbance, and the present and predicted scale of accelerated climate change and cascading ecologic effects in the North give urgency to the need for a better understanding of these systems now and in the future. These environmental changes may presage substantial consequences for health, economic well-being, and continuity of culture and society at a global level.

**Figure 1:**
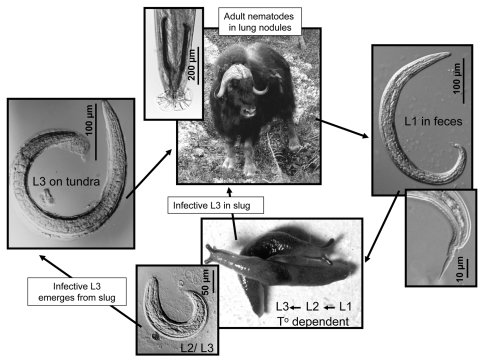
Life cycle of protostrongylid parasite: *Umingmakstrongylus pallikuukensis* in muskoxen definitive and gastropod intermediate hosts (*12*). Adult nematodes (for *U. pallikuukensis*, located in the lungs) lay eggs, which hatch to first-stage larvae (L1). L1 move up the airways, are swallowed, and pass in the feces, where they must invade the foot of gastropod intermediate hosts for further development to the infective third-stage larvae (L3). Development to L3 requires a minimum amount of heating and does not occur below a critical threshold; these development parameters vary among different protostrongylid species (*6*,*7*,*13*). Definitive hosts become infected by ingesting a gastropod containing L3 or, for some protostrongylids such as *U. pallikuukensis*, by ingesting L3 that have emerged from the gastropods and are free in the environment.

The current International Polar Year, which covers the period March 2007 to March 2009 (although officially designated International Polar Year 2007–2008), offers a unique opportunity to develop a baseline, or snapshot, of the North and to explore the patterns and processes for infectious disease emergence in northern ecosystems that are globally relevant. As a contribution to this baseline, we outline approaches, protocols, and empirical models derived from a decade of exploration of pathogen biodiversity and disease in northern ecosystems (Table 1), illustrated with examples of drivers for emerging parasitic disease in northern wildlife (Table 2). Categories of emergence explored in our studies include 1) geographic expansion, 2) host switching, 3) resurgence due to climate change, 4) new introduction, and 5) new recognition of pathogens. This approach can be viewed as a broadly applicable framework for defining pathogen diversity, including distinguishing what is truly emerging from what is newly discovered, and the historical and contemporary drivers associated with emerging infectious diseases in the North and elsewhere in the world. This ecologic approach to understanding disease emergence in wildlife complements a growing recognition of the need for long-term epidemiologic datasets to detect the effects of climate change on human health (*8*). Collectively, these constitute a critical sentinel for the interaction of climate and infectious disease in the global arena.

**Table 1:** Approaches and tools for exploring diversity and changes in complex host-parasite systems

Definition of pathogen diversity
1) Geographically extensive and site intensive survey and inventory
2) Determination of faunal diversity for species present, for which systematics is the foundation
3) Patterns of association for hosts
4) Geographic range for hosts and parasites
5) Numerical, abundance/ intensity data
6) Seasonal data for distribution and patterns of transmission
7) Survey linking parasite species diversity to population structure requiring integrated morphologic and molecular approaches for accurate and rapid diagnostics
8) Molecular prospecting for diversity
9) Distribution of parasites versus distribution of disease
Development of historical baselines
1) Archival museum collections
2) Host-parasite phylogenetic frameworks
3) Historical ecology and biogeography/phylogeography to clarify past abiotic and biotic determinants of distribution
4) Geographic information system applications
5) Analogue approaches to be applied where historical processes that have structured faunas are used to inform or predict the responses of contemporary systems under a regime of dynamic climate change
Exploration of environmental effects
1) Define thresholds and rates for development
2) Define tolerances for environmental parameters, e.g., temperature, humidity, precipitation
3) Define environmental limitations on distribution
Characterization of disease conditions
1) Laboratory-based experimental infections in parasite naïve hosts
2) Pathology and histology
3) Evaluations of natural mortality and associations with parasites
Establishment of surveillance networks and monitoring
1) Targeted strategic survey and inventory
2) Opportunistic networks linking wildlife managers and communities
Development and testing of predictive models
1) Integrative frameworks incorporating data from survey, parasite diversity, historical analogues, environmental thresholds, tolerances and constraints
2) Responses under scenarios for climatologic/environmental change
3) Validation through long-term monitoring

**Table 2:** Responses to climate warming and drivers for emergence of parasites and parasitic diseases in Arctic systems

Numerical responses (changes in abundance of parasites)
1) Temperature-mediated increases in rates of development for free-living stages, or those in intermediate hosts
2) Reduced parasite generation time, e.g., shifts from multiyear to single-year cycles, or from single to multiple within year
3) Environment-mediated changes (increases or decreases) in survival rates for developmental stages
4) Extension of season for parasite growth and transmission resulting from earlier thaw in spring and/or later freeze during fall
5) Amplification of parasite populations over time through accelerated development, increased rates of transmission, survival, and availability
6) Increases in parasite prevalence and abundance
7) Changes in density-dependant linkages for hosts and parasites leading to altered patterns of abundance for host populations
Functional responses (changes in host and geographic ranges)
1) Shifting patterns of geographic range for hosts and parasites including latitudinal and/or altitudinal shifts
2) Alterations in host range for parasites through geographic and host colonization, successful establishment in naive host species or host populations
3) Changing phenology (timing) for habitat use through alteration of migration and migratory corridors, relative changes in spatial and temporal overlap
4) Modification of ecotones and contact zones including northward or southward expansion for hosts and/or parasites if environmental tolerances are not exceeded
5) Local extirpation because conditions exceed developmental tolerances
Microevolutionary responses
1) Local adaptation through selection for optimal patterns of development
2) Directional changes in gene frequencies for parasites
3) Geographic mosaics or ephemeral patterns of local adaptation and emergence
Cumulative/synergistic responses
1) Breakdown in mechanisms for ecologic isolation promoting faunal interchange for hosts and parasites and cascading changes in ecosystems
2) Variable and cumulative synergy affecting the structure of entire parasite–host communities during episodes of climate change

## The Approach and Tools

### Defining Pathogen Diversity

**Figure 2:**
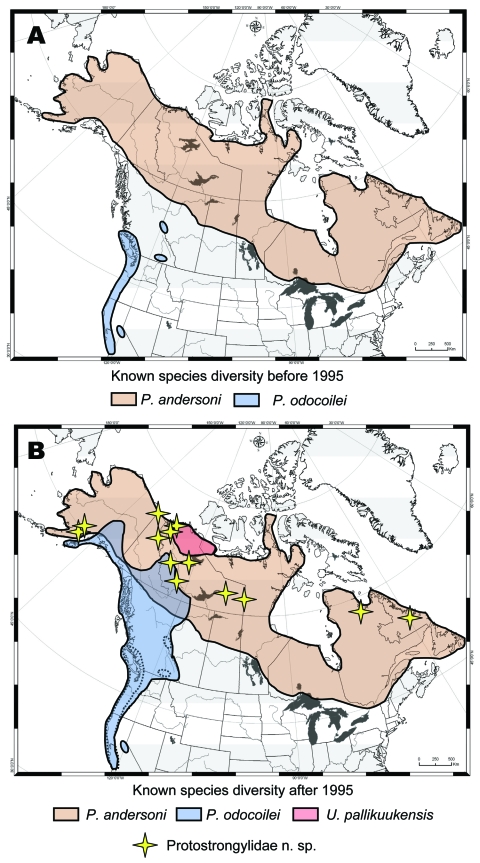
Geographic ranges for protostrongylid parasites in northern ungulates showing how survey and inventory have dramatically altered our understanding of diversity and distribution, before (A) and after (B) 1995. Distributions are depicted for *Parelaphostrongylus andersoni* in caribou (*19*,*20*); *P. odocoilei* in wild thinhorn sheep, mountain goat, woodland caribou, black-tailed deer, and mule deer (*15*,*17*); *Umingmakstrongylus pallikuukensis* in muskoxen (*12*,*14*); and a putative new species of Protostrongylidae in moose, caribou, and muskoxen (*20*). The range for *P. andersoni* in the North is presumed to coincide with caribou, although records substantiated by survey are few (*19*,*20*). Protostrongylids have not been detected in ungulates from the Arctic islands and Greenland and may be excluded from these high latitudes under current climate conditions.

Until recently, few baseline investigations had been carried out on the health of wildlife in northern North America (*4*,*14*–*18*). To develop an accurate and comprehensive baseline of diversity (parasite species and their host and geographic associations), we conducted opportunistic and targeted field surveys (Figure 2). Physical vouchers (representative specimens that are definitively identified and held in permanent museum repositories), frozen tissues, and associated data formed the basis for archival collections (*9*,*11*,*18*). Archival collections link surveys, informatics, phylogenies, history, biogeography, and ecology to form long-term baselines for exploring biodiversity and the evolutionary and ecologic determinants of infectious disease emergence (*3*,*9*,*18*). Such baselines allow tracking of changes in pathogen diversity and genetics, as well as differentiation between new detection versus true emergence of diseases.

Challenges associated with defining pathogen diversity in the North included a literature in which parasites of northern wildlife were often assumed to represent the same array of species as those commonly found in domestic animals from temperate regions. Further complications arose from the absence of properly preserved and documented parasite specimens and the specialized knowledge needed to identify such specimens to the species level. To identify parasite species in wildlife across extensive geographic ranges in remote northern locations, we developed molecular probes for the identification of larval parasites in feces. We used these techniques to redefine the distribution of a parasite (*Parelaphostrongylus odocoilei*) in multiple host species across northwestern North America (*15*) and to detect at least 1 new protostrongylid species in moose, muskoxen, and caribou in northern North America (*20*; Figure 2); these fell within the category of emergence of new recognition of a pathogen. In addition, by using a combination of morphologic and molecular methods of identification, we detected a host switch of a lungworm (*Protostrongylus stilesi*) from Dall sheep to sympatric muskoxen (*21*) (category of emergence: host switching).

A fine-scale understanding of these host and geographic associations allows us to identify naive wildlife populations and host species vulnerable to colonization by protostrongylids and other parasites. For example, exchange of protostrongylid parasites (*Umingmakstrongylus pallikuukensis* and *P. stilesi*) may occur between reciprocally naive populations of endemic and introduced subspecies of muskoxen at the ecotone (interface between >2 regions or habitats) formed by the Mackenzie River (*4*,*21*) (category of emergence: host switching and geographic expansion). In addition, our extensive survey across northern North America reinforces the hypothesis that Old World protostrongylids (i.e., species of *Elaphostrongylus*, *Neostrongylus*, and *Cystocaulus*) do not naturally occur in North America and have not yet been introduced (*20*), with the exception of *Elaphostrongylus rangiferi*, which has been translocated and established on the island of Newfoundland in eastern Canada (*19*) (category of emergence: new introduction of pathogen).

### History of Northern Host–Parasite Systems

The diversity and distribution of hosts and pathogens in the North have been structured by dynamic climate change during the past 3 million years through alternating cycles of glaciation/deglaciation and isolation/expansion (*10*,*22*). Increasingly cold climate during the Pliocene and Pleistocene periods may have further influenced selection of an Arctic-adapted fauna with its own unique developmental constraints and environmental tolerances (*10*,*22*). Historical climate dynamics can serve as an analog for the effects of accelerated change currently experienced in the North (*9*,*18*,*22*), including the emergence of pathogens and disease. Based on our understanding of the past determinants for northern host–parasite systems, we predict increased host switching and range expansion as climate change removes ecologic barriers and developmental constraints for pathogen transmission and redraws the maps of host distributions and timing of seasonal movements by hosts (*10*,*23*).

A broader understanding of the drivers for infectious disease emergence is in part dependent on appreciating why pathogens occur in particular regional settings and host groups, which is in turn determined by evolutionary and ecologic constraints that have structured pathogen diversity in space and time (*11,22,23*; Table 2). Our interpretations of current host and geographic distributions of northern parasites have been guided by a strong foundation in historical processes, both recent and deep (10 thousand years before present [KYBP] to 5 million years before present [MYBP]) (*9*,*18*,*22*). For example, the current focal distribution of the Arctic-adapted lungworm *U. pallikuukensis* in a circumscribed population of muskoxen (Figure 2) is attributed to recent host extirpation and reduction to a remnant population in the early 1900s, primarily as a result of hunting (*12*,*14*). In contrast, deeper historical processes (600–300 KYBP) account for widespread distribution of the lungworm *P. stilesi* in wild sheep, which entered North America across Beringia, the land mass that historically joined North America and Siberia (*10*,*24*). The muscleworm *P. odocoilei* has a more heterogeneous distribution in wild sheep because these hosts were colonized relatively recently (late Pleistocene) in a host-switching event from deer (*Odocoileus hemionus*), probably when the 2 species were concentrated in glacial refugia, and later expanded northwards following deglaciation (categories of emergence: host switching and geographic expansion) (*15,24*; I. Asmundsson, E.P. Hoberg, E.J. Jenkins, unpub. data).

Knowledge of phylogeny of pathogens and co-evolutionary processes for parasites and hosts provides powerful insights about life cycles, host specificity, and site specificity within the host and the potential for emergence (*3*,*11*,*22*,*23*). For example, knowledge of the relationship and behavior of related parasites provided immediate information on the likely life history of *U. pallikuukensis* (*12*,*14*) and site specificity and potential hosts for *P. odocoilei* (*17*) and is now guiding our search for adults of a previously undescribed protostrongylid (*20*). Finally, we have a unique window into the past to examine historical diversity and phylogeny as pathogens in frozen feces of caribou and other northern wildlife species (both extant and extinct) emerge from receding ice packs—a natural cryoarchive. Whether these ancient pathogens represent a risk for reemergence of disease remains undetermined (*25*).

### Investigation of Natural and Experimental Disease

New host and geographic records for a pathogen do not constitute an emerging infectious disease unless the pathogen, either alone or in combination with other factors (e.g., weather, malnutrition, predation), actually causes disease. Disease associated with macroparasites is often sublethal and difficult to detect in free-ranging wildlife. This is especially true in the North, where the sheer size and diversity of the geographic areas, low densities and isolation of human communities, and remote study areas hamper year-round observation of illness and death in wildlife. Therefore, we recruited local community members and hunters to create a network of “wildlife health monitors,” who are trained to collect standardized samples and data to develop baselines for wildlife health over time (http://wildlife1.usask.ca/sahtu/monitors.php). Even if a decline in a wildlife population is detected and documented, the role of disease can be difficult to tease from an array of complex ecologic relationships. Therefore, to isolate the effects of single pathogens and evaluate subclinical disease, we experimentally infected captive wildlife and monitored them by using a variety of veterinary clinical techniques (*24*,*26*).

Within the host, pathogenesis varies relative to parasite species, intensity of infection, and host factors such as age and immune status. In muskoxen, infections of *U. pallikuukensis* are cumulative with age, and adult parasites are associated with large, space-occupying lesions in the lungs. Infected animals exhibit epistaxis and may be more susceptible to predation (*12*,*14*). In wild sheep, *P. odocoilei* causes diffuse, pulmonary granulomas that can lead to respiratory failure in heavily infected or otherwise compromised animals. Experimentally infected sheep exhibited marked weight loss and transient neurologic signs, which, together with decreased respiratory function, would compromise their ability to escape predation at high altitudes and over rough terrain (*24*,*26*). Also, respiratory pathology may result from synergy of *P. odocoilei* with the lungworm *P. stilesi* because both parasites are present in almost all wild sheep in some regions (*24*,*26*). Infections of *Parelaphostrongylus andersoni*, possibly in combination with the new protostrongylid species, have been linked to verminous pneumonia in barren-ground caribou in Alaska and Canada.

The potential for protostrongylids to cause disease in individual hosts is apparent, but unequivocal evidence linking infection to declines in population health, numbers, or both remains to be demonstrated. Detection of population-level effects of protostrongylid parasites through experimental treatment of subpopulations is confounded by the broad-spectrum nature of anthelmintic treatment, which would eliminate many other nematode species (*27*). Therefore, future work may be limited to modeling the effects of these parasites on host populations and monitoring causes of illness and death in natural populations.

### Climate Change as a Driver for Infectious Disease Emergence

An evidence-based process is necessary to demonstrate a clear link between climate change and emergence of pathogens and disease, as the interactions are complex and nonclimate drivers may alter patterns of disease in both animals and humans (*2**,**8**,**28**,**29*; Table 2). To connect the dots, we must 1) collect baseline data on distribution, epidemiology, and effects of pathogens; 2) isolate the effects of temperature on pathogen transmission in laboratory and field; 3) provide regional evidence of climate change; 4) forecast temporal and spatial effects of climate change on parasite and host populations; and 5) detect epidemiologic and health consequences of climate change (*2*,*24*). In our study system, protostrongylid parasites in northern ungulates, we have accomplished much of steps 1–4 and continue to develop a detection system for step 5.

For step 1, an integrative research framework showed substantial new insights about the distribution, host associations, and behavior of pathogenic parasites in northern wildlife (Table 1). For step 2, field and laboratory experiments showed specific tolerances and thresholds and a direct relationship between temperature and development of *U. pallikuukensis* and *P. odocoilei* in intermediate hosts (*7*,*12*). For step 3, historical temperature data demonstrated that the core region for our studies (the Mackenzie District of the Northwest Territories) had the highest rate of warming in Canada (60-year warming trend of 2.2°C in annual temperature; www.msc-smc.ec.gc.ca/ccrm/bulletin/annual06/regional_e.cfm), and that warming available for parasite development had already increased at multiple sites in the Canadian Arctic (*4*,*6*,*7*). For step 4, a model for temperature and rates of larval development in gastropod intermediate hosts was validated for these protostrongylids and applied to climate warming scenarios (*6*,*7*). Based on these projections, we determined that climate warming led to substantial amplification of parasite populations in disease-endemic areas through reduction in generation times and broadened seasonal windows for transmission, contributing to heightened intensity of infection among individual hosts (*4*,*6*,*7*). Climate change also may result in release of some “generalist” parasites (e.g., *P. odocoilei*) from environmental constraints, which, in combination with a breakdown in mechanisms for ecologic isolation, could facilitate range expansion and colonization of naïve hosts (2 categories of emerging infectious disease) (*7*).

For step 5, we demonstrated that climate change precipitated a switch from a multiyear to single-year cycle of transmission for *U. pallikuukensis*, possibly starting in the late 1980s and coinciding with reports of clinically ill muskoxen in the disease-endemic region (category of emergence: resurgence due to climate change) (*4*,*6*). Following the warmest recorded annual temperature for this region in 1998 (www.msc-smc.ec.gc.ca/ccrm/bulletin/annual06/rsummarytable_e.html?table=temperature&season=annual&date=2006&nyears=59), fatal pneumonia associated with *P. odocoilei* was first detected in wild sheep in the Mackenzie Mountains in 1999, although this may reflect increased vigilance rather than true emergence. Emergence of disease may follow climate change, but for macroparasites in particular, there are likely to be lag times determined by a period of development in the host population (*30*). Cascading and cumulative long-term effects of climate change, including shifts in host–pathogen relationships, may be among the factors contributing to large-scale changes in abundance and distribution observed in keystone wildlife (e.g., boreal woodland and barren-ground caribou) in northern North America.

### Lessons Learned and the Way Forward

During the past decade, our investigations of emerging parasitic infections and diseases in northern wildlife have moved from opportunistic survey to targeted surveillance and hypothesis-driven research (Table 1). This work relied on field observation, laboratory experimentation, and enhanced diagnostic capacity by a network of collaborators with expertise in traditional parasitology, morphologic and molecular systematics, population genetics, epidemiology, wildlife biology, and traditional ecologic knowledge. Exchange of information relied on a system to collect and respond to community-based local knowledge and to respond to feedback in the form of community meetings and reports accessible to a lay audience. Meaningful engagement of aboriginal communities and integration of Western science with traditional knowledge has become a key part of any northern research (*31*; http://wildlife1.usask.ca/sahtu) and is a major focus of International Polar Year.

International Polar Year is a unique opportunity to address a critical need for baselines and archives needed to detect and explore drivers for infectious disease emergence in northern systems. While past efforts have been primarily opportunistic or specific to a particular issue, species, or location, we are seeing the development of long-term programs with a broad vision ensuring a comprehensive approach, such as the Beringian Coevolution Project (*9,18*; http://nix.msb.unm.edu; http://wildlife1.usask.ca/iwap/abstracts/galb.html) and the Circumarctic Rangifer Monitoring and Assessment Network (www.rangifer.net/carma/index.html; http://classic.ipy.org/development/eoi/proposal-details.php?id=162). Such programs depend on effective communication among research groups that have traditionally been on parallel but isolated trajectories; they also require a strong commitment to follow-up, often one of the biggest challenges in disease management and research on a global scale.

Another challenge is to recognize that neglected pathogens and legacy diseases, such as those caused by macroparasites, deserve equal research attention as “new” pathogens. Among sublethal effects of macroparasites are compromised physical and intellectual development and ability in humans (*32*) and reduced productivity and fecundity in domestic livestock (*33*). Examples of regulation of wildlife populations by macroparasites are increasing, often in combination with malnutrition or predation (*27*,*34*). Despite this, microparasites (generally prions, viruses, bacteria, fungi, and protozoans) often remain the singular focus of research on emerging infectious diseases (*35*,*36*). Greater effort needs to be dedicated to the effects of macroparasites on population health and demographics to ensure detection and proactive management of emerging diseases caused by these organisms. Our next challenge will be to understand the effects of interactions of multiple pathogens (both micro- and macroparasites), nutrition, stress, and other environmental factors on health of both individuals and populations.

Our work has resulted in a 10-year baseline for wildlife health across a region of northern North America increasingly vulnerable to climate change and other drivers of infectious disease emergence. Climate change will affect northern ecosystems by altering host range (i.e., expansions, shifts, increased or decreased overlap), abundance, and resilience, in addition to availability of environmental contaminants, free-living stages of pathogens, and intermediate hosts/vectors (Table 2). Climate change may also interact with other drivers for infectious disease emergence in northern North America. These include 1) development of new ecotones related to expansion of southern animal species, such as deer, red fox, and domestic livestock; 2) altered routes and timing of migration for wild birds and caribou herds; 3) habitat alteration and fragmentation due to resource extraction and development; and 4) historical and ongoing translocation of hosts and pathogens (e.g., historical introductions of reindeer and muskoxen, natural expansion of existing wood bison populations, proposed reestablishment of wood bison in Alaska, and increasing interest in livestock production in the North). The cumulative implications for health of wildlife that are culturally and economically important for northern communities are unlikely to be positive (*37*). In addition, environmental change is predicted to drive increased dissemination of zoonotic pathogens in water- and foodborne pathways (e.g., *Giardia*, *Cryptosporidium, Toxoplasma, Trichinella,* and *Echinococcus* spp.), posing a direct threat to human health in communities that rely on local water sources and country foods; environmental change may also lead to emergence of disease resulting from “spillover” of pathogens between persons and wildlife (*8*,*16*,*37*–*39*).

Effects of climate change on infectious disease emergence may be cumulative and play out over decades or may manifest as isolated and extreme events. Long-term cumulative processes may be the drivers for changing dynamics between hosts and pathogens (e.g., generation time, developmental rates, amplification, shifts in seasonal transmission) and create subtle effects that will be challenging to demonstrate in the absence of extensive baselines (*4*,*6*,*7*). Incremental and gradual increases in temperature may drive thresholds or tipping points influencing the interface for hosts and parasites (*6*). In contrast, extreme weather events of temperature and humidity, as an outcome of climate change, are predicted to result in a mosaic of ephemeral or explosive emergence of disease against this broader background. Signals for these patterns have already been demonstrated in the Arctic (*30*,*40*).

We seek to emphasize the generalities for processes of emergence for both animal and human pathogens, and offer an approach that may serve as a universal framework to understand, anticipate, and forecast change in these complex systems on global and regional scales. These drivers are not unique to the North; they are in action in scenarios all over the world (*8*). Wildlife pathogens, or diseases originating in wildlife, are emerging as a considerable threat to human health worldwide (e.g., severe acute respiratory syndrome, HIV/AIDS, Ebola, Hendra, Nipah, and avian influenza viruses) (*2,23*; www.cws-scf.ec.gc.ca/cnwds/intro_e.cfm). While our work focused on a nonzoonotic group of pathogenic nematodes, our integrated, multidisciplinary approach can be extrapolated to investigate a broader diversity of pathogens in the North and anywhere else where the animal-human interface is intact or expanding (Table 1). The North has emerged as a region of great interest because of the historical interaction between people and wildlife, the current impact of climate change on northern ecosystems, and the future importance of the natural resources (both renewable and nonrenewable) in this region.
